# Biomarker microRNA-371a-3p - expression in malignancies other than germ-cell tumours

**DOI:** 10.1007/s00432-025-06101-4

**Published:** 2025-01-31

**Authors:** Gazanfer Belge, Markus Klemke, Bendix Hansen, Cansu Dumlupinar, Aylin Igde, Dirk Arnold, Hans Salwender, Christian Wülfing, Armin Soave, Klaus-Peter Dieckmann

**Affiliations:** 1 Department of Tumour Genetics, Faculty of Biology and Chemistry, University of Bremen, Bremen, Germany; 2 mir|detect GmbH, Bremerhaven, Germany; 3 Department of Urology, Asklepios Tumorzentrum Hamburg, AK Altona, Hamburg, Germany; 4 Department of Oncology, Asklepios Tumorzentrum Hamburg, AK Altona, Hamburg, Germany; 5 Department of Urology, University Medical Center Hamburg-Eppendorf, Hamburg, Germany

**Keywords:** Testicular germ cell tumour, Biomarker, microRNA-371a-3p, Multiple myeloma, Chromosome 19, test specificity

## Abstract

**Purpose:**

microRNA-371a-3p (M371) is considered a highly sensitive and specific serum biomarker of testicular germ cell tumours (GCTs). However, little is known about the expression of M371 in nontesticular malignancies (NTMs), so far. As knowledge about the expression of the marker in other malignancies is a prerequisite for the clinical application of the test we aimed to explore the M371 expression in other cancers.

**Methods:**

M371 serum levels were measured in 137 patients with NTM of 12 different neoplastic entities using the IVDR-certified M371-Test for quantitative real-time PCR. Median M371 serum levels and percentages of M371 level elevations were calculated for the entire NTM group and for entity-specific subgroups. The results were compared with GCT patients (*n* = 20) and with tumour-free male controls (*n* = 20) using descriptive statistical methods.

**Results:**

Eight patients with NTMs had M371 serum level elevations, corresponding to a false-positive rate (FPR) of 5.84% (95% confidence intervals (CIs) 2.55–11.18%). Expression rates in GCTs and controls were 100% and zero, respectively. Thus, the specificity of the M371-Test for GCT is 94.90% (95% CI 90.21–97.77%) when all NTMs and tumour-free controls are considered. Remarkably, three out of 5 patients with multiple myeloma had elevated M371 levels.

**Conclusion:**

The false-positive rate of the M371-Test in other malignancies than GCT is very low, and almost identical with that in healthy males, corresponding to a high specificity of 94.9% for detection of GCT. The surprising finding of M371 elevations in patients with multiple myeloma needs further investigation.

## Introduction

During the last decade, microRNAs (miRs) of the miR-371-373 cluster and their putative roles in the clinical management of testicular germ cell tumours (GCTs) have been extensively studied (Leão et al. 2021; Seales et al. 2024). In particular, serum levels of microRNA-371a-3p (M371) were shown to feature many of the characteristics a valuable biomarker is supposed to have according to the criteria stipulated by Lange and Winfield in 1987 (Lange and Winfield 1987). M371 involves 90% sensitivity and > 90% specificity for primary diagnosis of GCTs (Dieckmann et al. 2017a, b). Likewise, relapses during surveillance of clinical stage 1 GCTs can safely be diagnosed (Lobo et al. 2021; Fankhauser et al. 2022; Belge et al. 2024), and the same is true for predicting histology of lymph nodes excised upon retroperitoneal lymph node dissection (Lafin et al. 2020; Seelemeyer et al. 2024). Also, the half-life of M371 after resection of GCT was shown to be less than 24 h (Radtke et al. 2018). Thus, M371 was considered to represent a novel and powerful serum biomarker of GCT eclipsing the traditional markers.

However, in spite of the multitude of studies conducted to explore the features of M371, only very few investigations have addressed the condition #1 of the Lange & Winfield list, i.e. the specification that the marker substance is only produced by the malignancy itself. Few reports documented the non-expression of M371 in testicular neoplasms of non-germ cell origin, such as sex cord gonadal stromal tumours, epidermoid cysts and malignant testicular lymphoma (Syring et al. 2015; van Agthoven and Looijenga 2017; Belge et al. 2020). Malignancies arising from other sites than the gonads have been evaluated in only one study to date (Spiekermann et al. 2015). That pilot study analysed 24 nontesticular malignancies and found zero expression of M371 in 12 cases but remarkably, it also noted traces of the miR in the other half of patients. Other studies revealed a physiological role of miR-371a-3p in regulating antiproliferative pathways via the TOB1 axis in colorectal and gastric cancer (Guo et al. 2019; Wang et al. 2020). Moreover, miRs of the miR-371-3a cluster have been found to be involved in molecular processes in tumorigenesis in various neoplastic diseases (Shah et al. 2021). Therefore, elevations of miR-371 serum levels in malignancies other than GCT are principally conceivable. Importantly, if elevations of M371 serum levels should be detectable in a relevant number of patients with other cancers, this finding could have a considerable bearing on the utility of M371 as biomarker of GCTs. To address this open issue, we analysed M371 serum levels in a large number of patients with various cancers and compared the results with measurements in both GCT patients and in healthy males.

## Patients, methods

### Patients, controls

Consecutive patients treated for nontesticular malignancies (NTMs) in the departments of haematology/ oncology and of urology in Asklepios Klinik Altona, Hamburg, during 2023–2024 were included. Fifty-one (37%) patients were examined before the start of curative treatment. The remainder were under treatment at the time of examination but far from cure. So, all patients included in the study were loaded with significant tumour masses at the time of examination. Treatment consisted of surgery in most of the urological cancers and in testicular lymphomas while chemotherapy with various regimens was applied in all others. Patient age and histologic tumour type were registered in each case. For comparison two control groups were included, first, patients with histologically proven GCT clinical stage 1 treated in the department of urology in Asklepios Klinik Altona 2024, and second, tumour-free male controls seeking general health check in the same institution during 2023–2024.

Informed consent was obtained from all individual participants included in the study. The Ethical Committee of Ärztekammer Bremen approved the study (HR/RE-301 A). All study activities followed the rules of the Declaration of Helsinki documented by the 64th General Assembly of the World Medical Association in October 2013.

### Laboratory methods

Cubital vein whole-blood samples were processed to serum by centrifugation at 2,500 g /10 minutes. Serum aliquots were then stored at minus 80 °C until further processing. Measurement of levels of microRNA-371a-3p were performed as described earlier, using the IVDR- certified M371-Test (mir|detect, Bremerhaven, Germany) for quantitative real time polymerase chain reaction (qPCR) and with miR-30b-5p as endogenous control (Dieckmann et al. 2019). Results were obtained as relative quantities (RQ values) according to the ∆∆Ct method (Livak and Schmittgen 2001). The standard cut-off used for primary diagnosis of GCTs, the value of RQ = 5, was also employed as upper limit of norm in this evaluation.

### Statistical analysis

Any M371 elevation above cut-off in NMT patients and controls was considered false-positive. Median values with interquartile ranges (IQRs) of M371 serum levels were calculated in GCT patients, healthy controls, in the entire group of NTMs, and in subgroups with specific neoplastic entities. Likewise, false-positive rates (FPR) with 95% confidence intervals (CIs) were calculated for each subgroup. Mann-Whitney U tests were performed to compare the relative expression (RQ-levels) of miR-371a-3p between subgroups and controls. Statistical significance was assumed at *p* < 0.05. Statistical analysis was performed using IBM SPSS Statistics for Windows, Version 29.0.2.0 (IBM Corp., Armonk, NY).

## Results

**Fig. 1:**
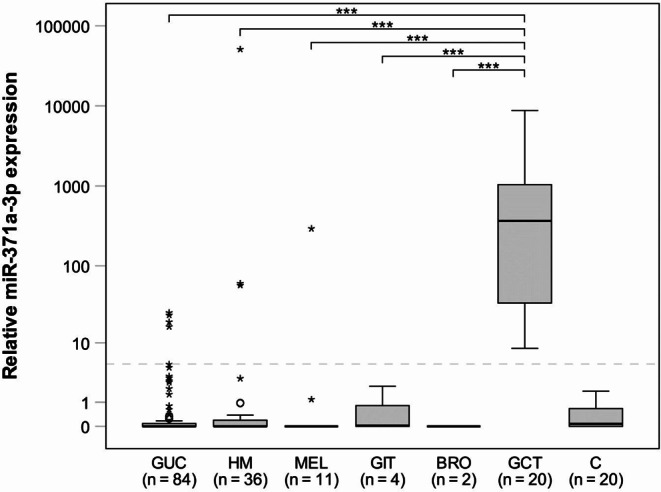
miR-371a-3p expression in testicular germ cell tumours, male controls and other malignancies. Median M371 expression in testicular germ cell tumours (GCTs) is significantly higher than that of controls and of each of the nontesticular malignancies. Box plot showing median miR-371a-3p expression (RQ value) in testicular germ cell tumours (GCTs), male controls and 137 other malignancies. The boxes display the first quartile, median and third quartile. The whiskers are defined as the largest or lowest observed value that falls within 1.5 times the interquartile range measured from Q3 or Q1. Mild outliers are represented by dots and extreme outliers by asterisks. The dashed grey line indicates the cut-off value (RQ = 5). The y-axis is depicted in a logarithmic scale. Significant differences are indicated by bars above the groups, *** *p* < 0.001. GUC: genitourinary cancers except GCT; HM: haematological malignancies; MEL: malignant melanomas; GIT: gastrointestinal tumours; BRO: bronchial carcinomas; C: tumour-free male controls

**Fig. 2:**
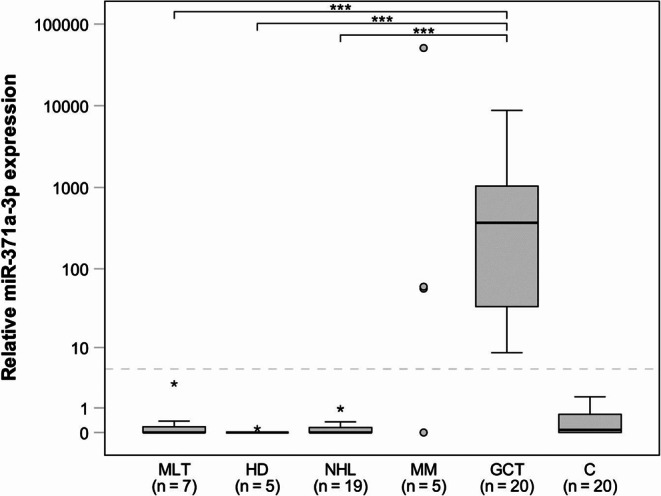
miR-371a-3p expression in serum of various subtypes of haematological malignancies in comparison to germ cell tumours and tumour-free male controls. Median M371 serum levels of patients with various subtypes of haematological malignancies are in the range of male controls and are significantly lower than the M371 level of testicular germ cell tumours. By contrast, no significant difference was observed between multiple myelomas and GCT. Box plot as outlined in Fig. 1. Significant differences are indicated by bars above the groups, *** *p* < 0.001. Extreme outliers are represented by asterisks. The dashed grey line indicates the cut-off value (RQ = 5). Because of extreme variance of results, the data in multiple myelomas are not given in a box-whisker format but as dots representing individual RQ values. The y-axis is depicted in a logarithmic scale. MLT: malignant testicular lymphoma; HD: Hodgkin´s disease; NHL: Non-Hodgkin lymphoma; MM: multiple myeloma; GCT: testicular germ cell tumour; C: tumour-free male controls

A total of 137 patients with 12 different NTMs were included, thereof 84 genitourinary cancers (GUCs), 36 haematological malignancies, 11 melanomas, 4 gastrointestinal cancers, and 2 bronchial carcinomas. Control groups consisted of 20 GCT patients, clinical stage 1, and 20 tumour-free males (Table 1). Over-all, 8 patients (FPR: 5.84%; 95% CIs 2.55–11.18%) with NTM were found to have M371 serum level elevations above cut-off, opposed to 100% in GCT patients and none in male controls. Tabulation of the separate measurement results of all malignant entities (Table 2) revealed that each one elevation was found in melanoma (among 11); prostate carcinoma (among 27); renal cell carcinoma (among 17), and two elevations in urothelial carcinoma (among 32). Strikingly, 3 out of 5 patients with multiple myeloma had elevations (FPR: 60%). Most of the serum level elevations in NTMs were only of modest extent with RQ-values of 16.51–59.97 (Figs. 1 and 2). However, one case with multiple myeloma revealed a considerable elevation of RQ = 50,920, surpassing even the highest values found in GCT patients (Fig. 2). To rule out laboratory technical failures, all of the RQ-values in multiple myeloma were confirmed by remeasuring. In addition, all three cases with M371 elevations were re-examined clinically, to rule out an occult testicular tumour. Statistical comparisons of RQ-values of the various subgroups with that of GCTs revealed significant differences with regard to each of the malignant entities tested, except for the group of multiple myelomas. The specificity for GCTs of the M371-Test is 94.16% (95% CI 88.82–97.45%) when the results of all malignancies other than germ cell tumours (*n* = 137) are considered and it is 94.90% (95% CI 90.21–97.77%) when all non-GCT samples, including tumour-free male controls (*n* = 157) are taken into account.

**Table 1:** Study population. Number and median age of patients included in study

Entity	(*n*)	Median age (years)	IQR (years)
**Genitourinary cancers**	84		
Prostate cancer	27	73.0	69–78
Urothelial carcinoma	32	69.0	60-79.75
Renal cell cancer	17	62.0	59-70.5
Penile cancer	3	52.0	
Spermatic cord sarcoma	5	60.0	41-70.5
**Haematological malignancies**	36		
Testicular malignant lymphoma	7	62.0	56–68
Hodgkin´s disease	5	49.0	32.5–73
Non-Hodgkin Lymphoma	19	66.0	54–77
Multiple myeloma	5	63.0	47.5–68
**Malignant melanoma**	11	n. a.	n. a.
**Gastrointestinal carcinoma**	4	58.5	33.25–79.25
**Bronchial carcinoma**	2	65.0	
**Germ cell tumour**	20	33.5	26.75–40.75
**Tumour-free male controls**	20	37.5	28-51.75

n. a.: not available

**Table 2:** Study results. False-positive rates and median M371 serum levels in subgroups

Entity	Total samples	Elevated M371-levels	FPR (%)	Median RQ-value (range)
**Genitourinary cancers**	**84**	**4**	**4.76**	**-**
Prostate cancer	27	1	3.70	0 (0.00–25.20)
Urothelial carcinoma	32	2	6.25	0 (0.00–23.46)
Renal cell cancer	17	1	5.88	0 (0.00–16.51)
Penile cancer	3	0	0.00	0.07 (0.00–0.24)
Spermatic cord sarcoma	5	0	0.00	0 (0.00–0.00)
**Haematological malignancies**	**36**	**3**	**8.33**	**-**
Testicular malignant lymphoma	7	0	0.00	0 (0.00–2.96)
Hodgkin´s disease	5	0	0.00	0 (0.00–0.12)
Non-Hodgkin Lymphoma	19	0	0.00	0 (0.00–0.96)
Multiple myeloma	5	3	60.00	56.68 (0.00–50,920.2)
**Malignant melanoma**	**11**	**1**	**9.09**	**0 (0.00–292.84)**
**Gastrointestinal carcinoma**	**4**	**0**	**0.00**	**0.01 (0.00–2.17)**
**Bronchial carcinoma**	**2**	**0**	**0.00**	**0 (0.00–0.00)**
**Germ cell tumour**	**20**	**20**	**n. a.**	**367.17 (8.46–8779.96)**
**Tumour-free male controls**	**20**	**0**	**0.00**	**0.07 (0.00–1.73)**
**All malignancies except GCT**	**137**	**8**	**5.84**	**-**
**All non-GCT samples**	**157**	**8**	**5.10**	**-**

FPR: false-positive rate

## Discussion

The crucial result of the present study is that a small proportion of 5.8% of patients with a variety of malignant nontesticular tumours have elevated M371 serum levels. This result is widely in line with the pilot study of 2015 (Spiekermann et al. 2015). The finding of a 94.9% specificity calculated from the data of the present study, underlines the great utility of M371 as a highly specific serum biomarker of GCT.

A rather startling finding is the high M371 expression in three patients with multiple myeloma. False-positive measurements can be ruled out because the RQ-values are rather high, in one case even higher than in GCT patients. Also, remeasurements confirmed the results precluding a technical failure. The underlying reasons for M371 expression in a high number of patients with multiple myeloma remain elusive. Statistical artifact based on the small number has to be taken into account, but again, this assumption is unlikely because of the rather high M371 levels found in these patients. Putatively, cytogenetic anomalies frequently encountered in multiple myeloma might represent the clue for understanding the unexpected finding. Cytogenetic analyses have revealed structural changes of chromosomes in 51% of cases with multiple myeloma (Laï et al. 1995). Chromosomal disorders involve losses of chromosomal material and also gains which may occur on several chromosomes, (Mohamed et al. 2007). Among others, chromosome 19 has been found to gain material in patients with multiple myeloma, and the chromosomal region 19q13 was found to be a typical breakpoint region in translocation processes (Nilsson et al. 2003). Importantly, the microRNA-cluster miR-371-3 is located on the long arm of chromosome 19 (19q13.4) in immediate vicinity to the breakpoint region. Thus, it appears conceivable that due to chromosomal rearrangements, the miR 371-3 cluster has been activated in at least a certain number of patients with multiple myeloma. Similar observations have already been made in thyroid adenomas (Rippe et al. 2010).

Clearly, the hypothesis that M371 serum level elevation in patients with multiple myeloma may be caused by cancer-related chromosome 19 alterations remains unproven because we do not have information on cytogenetic analyses in these patients. As yet, the frequency of that event is not clear, because only 5 patients have been evaluated so far. Further, any associations of M371 expression with oncological stages and other clinical features remain unknown. Nonetheless, 3 out of 5 patients having elevated expression of M371 appears to be more than a chance finding and consecutively, a possible expression of M371 in a number of patients with multiple myeloma should be considered upon clinical application of the test. Further investigation of elevated M371 levels in multiple myeloma patients is required preferably with employing digital-droplet PCR which could afford a more precise measurement than the PCR method used herein.

If multiple myeloma is excluded from the analysis, the relative frequency of M371 expression in NTMs would only be 3.8% (5/132). This frequency is still somewhat higher than the zero expression rate found in tumour-free controls in the present study. However, this null finding is probably related to the small sample size of controls. Low frequencies of M371 level elevations in healthy males (around 3–5%) had been found in two previous studies (Dieckmann et al. 2017a, b, 2019) where considerably larger control groups were employed. Accordingly, there is settled evidence for serum level elevations of this miR in very few individuals of the healthy male population. The 3.8% frequency in nontesticular cancers found herein, closely aligns with M371 elevations found in healthy controls previously. In consequence, false-positive rates are obviously almost identical in healthy controls and in other malignancies. This view is probably underlined by the only modest extents of M371 serum level elevations in NTMs. The base-line M371 expression rate of < 5% in NTMs (with multiple myeloma excluded) appears to be of little clinical relevance.

The very low false-positive rate of M371 documented in the present series unveils some minor analogies with the established serum tumour markers of GCT, human beta chorionic gonadotropin (bHCG) and alpha fetoprotein (AFP). Both of the traditional marker substances are likewise expressed in several nontesticular health conditions. In particular, they are highly elevated in maternal serum during early pregnancy, and accordingly, bHCG and AFP are routinely used for monitoring pregnancy. Furthermore, bHCG is highly elevated in gestational trophoblastic neoplasia, and sporadic elevations occur in a number of other cancers (Pedrazzoli et al. 2021). False-positive results may occur in the presence of high levels of luteinizing hormone (LH) (Takizawa et al. 2017). AFP is specifically associated with hepatocellular carcinoma and may be elevated in other hepatic diseases as well (Lani et al. 2024). Also, around 2% of healthy males were found to have slightly elevated levels of AFP in the absence of any disease (Dieckmann et al. 2017a, b).

Limitations of this study mainly relate to the small number of cases with multiple myeloma and to the lack of information regarding oncological and clinical details of the cases. Control groups are still small, too, and the median age of healthy controls is considerably lower than that of patients with nontesticular malignancies. Information on serum levels of bHCG and AFP is missing. Thus, direct comparison of M371 with traditional markers is not possible.

## Conclusions

The M371 expression rate in serum of patients with other cancers is in the range of that in healthy males. Thus, the first condition of the Lange & Winfield criteria for valuable tumour markers is clearly fulfilled by the M371-Test (Lange and Winfield 1987). This miR represents a serum biomarker with an outstandingly high specificity for germ cell tumours. There is a first indication for the expression of M371 in patients with multiple myeloma. This novel information warrants further investigation including evaluation of other miRs of the cluster.

## Data Availability

The datasets analyzed during the current study are available from the corresponding author on reasonable request.
